# Whether Primary Bone‐Only Oligometastatic Nasopharyngeal Carcinoma Patients Benefit From Radiotherapy to the Bones on the Basis of Palliative Chemotherapy Plus Locoregional Radiotherapy?—A Large‐Cohort Retrospective Study

**DOI:** 10.1002/cam4.70315

**Published:** 2024-11-04

**Authors:** Wan‐Ping Guo, Guo‐Dong Jia, Si‐Yi Xie, Xuan Yu, Xiao‐Han Meng, Lin‐Quan Tang, Xiao‐Yun Li, Dong‐Hua Luo

**Affiliations:** ^1^ Department of Nasopharyngeal Carcinoma State Key Laboratory of Oncology in South China, Guangdong Key Laboratory of Nasopharyngeal Carcinoma Diagnosis and Therapy, Guangdong Provincial Clinical Research Center for Cancer, Sun Yat‐Sen University Cancer Center Guangzhou China

**Keywords:** bone metastasis, nasopharyngeal carcinoma, oligometastases, prognosis, radiotherapy

## Abstract

**Objectives:**

Whether to perform local radiotherapy on metastatic bone for primary bone‐only oligometastatic nasopharyngeal carcinoma (NPC) patients remains unclear. Therefore, we analyzed the treatment methods and their survival and developed a prognostic model to predict outcomes and guide personalized treatment.

**Materials and Methods:**

We studied 308 primary bone‐only oligometastatic NPC patients who were treated with either palliative chemotherapy (PCT) alone, PCT combined with locoregional radiotherapy (LRRT), or PCT, LRRT, and radiotherapy to metastatic bones (bRT). The primary endpoint was overall survival (OS). Cox regression was utilized to identify independent prognostic factors, leading to the construction of a nomogram model. Patients were stratified into two risk groups based on median prognostic scores, and treatment modalities were compared using log‐rank test while employing the inverse probability of treatment weighting (IPTW) to balance baseline characteristics and adjust for sample size differences between risk groups.

**Results:**

The best OS was observed in the group treated with PCT, LRRT, and bRT (HR = 0.60, 95% CI: 0.45–0.81, *p* = 0.002). Multivariable analysis revealed that age, N stage, pre‐treatment levels of LDH, and EBV DNA were independent prognostic factors for OS. In total, 155 patients were in low‐risk group while 153 were in high‐risk group. Before and after IPTW, the high‐risk group benefited from the PCT, LRRT, and bRT regimen (adjusted HR = 0.53, 95% CI: 0.42–0.67, *p* < 0.001; unadjusted HR = 0.59, 95% CI: 0.42–0.83, *p* = 0.007), while the low‐risk group did not (adjusted HR = 0.79, 95% CI: 0.56–1.11, *p* = 0.345; unadjusted HR = 0.65, 95% CI: 0.37–1.14, *p* = 0.309).

**Conclusion:**

Best outcomes of the whole cohort were seen with PCT + LRRT + bRT. Our study identified age, N stage, pre‐treatment LDH levels, and EBV DNA levels as independent prognostic factors for OS. The high‐risk group demonstrated a longer OS when treated with PCT + LRRT + bRT, whereas the low‐risk group did not benefit from the combinatorial treatment.

## 1 Introduction

Nasopharyngeal carcinoma (NPC) originates from the nasopharyngeal epithelium and has an extremely unbalanced geographical distribution, with a high incidence in Southeast Asia [1, 2, 3]. Due to the insidious location of the nasopharynx, over half NPC patients are diagnosed at advanced stages. Nearly 10% have distant metastases at initial diagnosis [4, 5]. Bone metastasis (BM), especially in the spinal bones, is frequently observed and accounts for more than 50% of all metastatic sites [6]. The survival of patients with BM varies considerably, with a median overall survival (OS) of 20.3–36.9 months [7, 8, 9, 10, 11], but they generally fare better than those with liver or multi‐organ metastases [12, 13], as some studies have indicated that patients with oligometastases have much longer OS [9, 14].

Primary bone‐only oligometastatic NPC, defined as the presence of no more than five bone metastatic foci at the time of diagnosis, has a better chance of long‐term survival or even a cure. This highlights the importance of tailored treatment approaches, including systemic therapy, locoregional radiotherapy (LRRT), and local radiotherapy (RT) to metastatic bone [7, 15, 16, 17, 18, 19]. Although treatment guidelines for oligometastatic patients provide valuable insights, the variability in recommendations can be confusing [20]. Systemic therapy followed by LRRT has emerged as a mainstream treatment for patients with de novo metastasis, especially those responding well to systemic therapy [21, 22, 23, 24]. However, ongoing research has yet to reach a consensus on the combined use of local treatment for metastases based on systemic therapy and LRRT [6, 25, 26]. Thus, optimal management strategies for oligometastatic NPC patients remain to be identified. Due to the scarcity of primary bone‐only oligometastatic NPC patients, evidence supporting RT for bone metastases is limited [7, 10, 15, 27]. Therefore, it is necessary to investigate the potential benefits of local treatment in bone‐only oligometastatic NPC and identify suitable candidates. This study retrospectively analyzed treatment methods and prognosis of these patients, developing a prognostic model to predict overall survival (OS) and guide individualized treatment.

## 2 Methods

### 2.1 Patients

**FIGURE 1:**
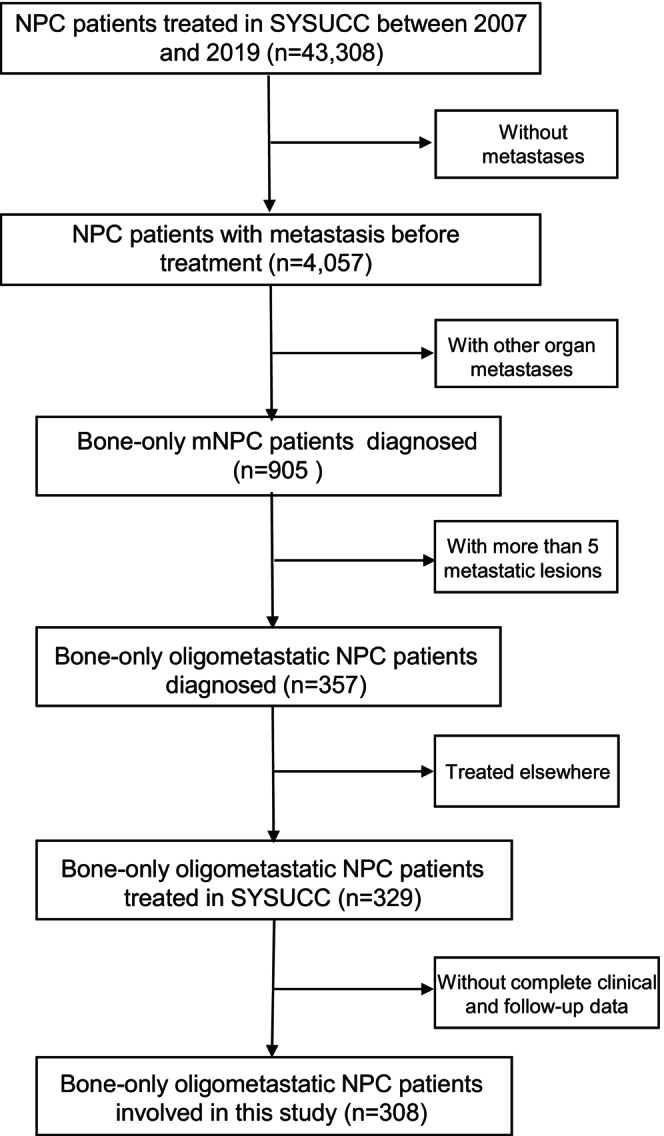
Diagram depicting the patient's inclusion flow. mNPC, metastatic nasopharyngeal carcinoma; NPC, nasopharyngeal carcinoma; SYSUCC, Sun Yat‐sen University Cancer Center.

We collected data of mNPC patients treated at Sun Yat‐sen University Cancer Center (SYSUCC) between September 2007 and December 2019, focusing on those with primary bone‐only oligometastases. Inclusion criteria included the following: (1) confirmed NPC diagnosis; (2) ≤ 5 de novo BM lesions; (3) received chemotherapy for at least four to six cycles; (4) a minimum follow‐up of 3 months; (5) Karnofsky performance status (KPS) ≥ 70; and (6) adequate organ functions (white blood cell > 4.0 × 10^9^/L; neutrophil > 2.0 × 10^9^/L; hemoglobin > 90 g/L; platelet > 100 × 10^9^/L; aspartate aminotransferase/alanine transaminase < 2.5 upper limit of normal; Ccr > 60 mL/min). Exclusions were coexisting or suspicious metastases to organs other than bone, coexisting pregnancy, lactation, or prior malignancies. A study flow chart is in Figure 1. Patients were restaged per the 8th American Joint Committee on Cancer (AJCC) TNM classification [28]. The study was ethics‐approved by SYSUCC (No. B2023‐575‐01).

### 2.2 Examinations

This retrospective study included all patients with complete medical records and follow‐up data. Patients underwent comprehensive medical history taking, physical examinations, magnetic resonance imaging (MRI) with contrast of the head and neck, and nasopharyngoscopy before treatment. Metastasis was verified by positron emission tomography‐computed tomography (PET‐CT) or whole‐body enhanced CT and bone scan. If bone lesions were suspected, contrast‐enhanced MRI was performed. All patients received quarterly follow‐ups for the first 3 years, biannual follow‐ups for at least 5 years, and then annual follow‐ups until death. Follow‐up examinations included nasopharyngoscopy, enhanced MRI of the head and neck, enhanced MRI of the metastatic sites, abdominal sonography or CT, chest radiography or CT, and plasma Epstein–Barr virus deoxyribonucleic acid (EBV DNA) measurements. Bone scans or PET‐CT were performed if recurrence or metastasis was suspected.

### 2.3 Treatment

Palliative chemotherapy (PCT) was administered as first‐line treatment to all patients. PCT was given every 3 weeks for four to six cycles using cisplatin‐based regimens: PF, cisplatin combined with 5‐fluorouracil; GP, cisplatin with gemcitabine; TPF, paclitaxel or docetaxel plus cisplatin and 5‐fluorouracil; and TP, docetaxel or paclitaxel plus cisplatin.

Some patients underwent RT including LRRT with or without bone radiotherapy (bRT) after PCT. LRRT is defined as RT to the primary tumor and cervical region. For LRRT, patients underwent intensity‐modulated radiotherapy (IMRT) or tomotherapy (TOMO) with a total dose of 66–72 Gy administered in 28–33 fractions to the primary lesion, 64–70 Gy administered in 28–33 fractions to the cervical lymph nodes, 60–63 Gy administered in 28–33 fractions to the high‐risk clinical target volume (CTV1), and 54–56 Gy administered in 28–33 fractions to the low‐risk clinical target volume (CTV2). IMRT or TOMO was administered once per day in five fractions per week for about 7 weeks. bRT refers to RT to the BM. The radiation methods used for BM include IMRT, TOMO, and stereotactic body radiation therapy (SBRT). For radiation to the BM site, a range of RT prescriptions were given to patients, and the common dose and fractionation regimens were 30 Gy/15f, 45 Gy/30f, 60 Gy/30f, etc. TOMO was administered to patients in whom RT of the primary and metastatic foci could be administered in one plan. If separate RT plans were administered, LRRT was administered first, followed by RT to the BM. The delivery of LRRT or LRRT + bRT was determined based on overall consideration of factors like BM quantity and location, overall performance status, age, treatment tolerance, and post‐PCT tumor response. For example, a patient who was young, had no other underlying disease, or had a markable reduction in EBV DNA load after PCT may receive the LRRT or LRRT + bRT even though PR was not achieved after PCT.

Concurrent chemotherapy (CCT) was administered to some patients during RT, with cisplatin dosages as follows: 30–40 mg/m^2^ weekly up to seven cycles or 80–100 mg/m^2^ tri‐weekly up to three cycles, starting on Day 1 of LRRT. Zoledronic acid or denosumab was given intravenously every 4 weeks once BM was confirmed, unless contraindicated. Secondary treatments including palliative chemotherapy, targeted therapy or immunotherapy, as well as participation in clinical trials or best supportive care, were considered for those showing recurrence or additional metastases.

### 2.4 Statistical Analyses

The primary endpoint was OS, defined as the time from diagnosis to death by any cause. The secondary endpoint, progression‐free survival (PFS), was defined as the period from diagnosis to progression or any‐cause death. PCT efficacy was assessed by Response Evaluation Criteria in Solid Tumors (RECIST) v1.1. Prior to statistical analysis, continuous variables like pre‐treatment lactate dehydrogenase (LDH) and alkaline phosphatase (ALP) levels were categorized using reference cutoffs of 250 U/L and 110 U/L, respectively. The cutoff for EBV DNA (10,000 copies/mL) was determined based on previous studies [29] and validated using a receiver operating characteristic (ROC) curve. And the cutoff for number of metastatic lesions (three) [7, 15] were determined by previous literatures. Metastatic bone sites were categorized as spinal, pelvic, sternum, ribs, or other regions. Bone destruction was categorized as either osteolytic or osteogenic. Osteolytic lesions were defined by trabecular destruction or reduced density, while osteogenic lesions were characterized by increased density or sclerotic changes.

Statistical analyses were performed using SPSS version 26.0 (SPSS Inc., Chicago, IL, USA) and R software version 4.2.1 (http://www.r‐project.org/). We used Kaplan–Meier curves to demonstrate OS and PFS, and inter‐subgroup differences were compared using the log‐rank test. The Cox model was employed for both univariate and multivariate analyses and to calculate the adjusted hazard ratio (HR) with a 95% confidence interval (CI) for independent OS prognostic factors.

A prognostic nomogram predicting 3‐ and 5‐year OS was developed based on independent risk factors from multivariate analyses. Discrimination and calibration of the nomogram were investigated using Harrell's concordance index (C‐index), a calibration plot with 1000 bootstrap resampling, and the area under the curve (AUC) of the ROC analysis. Discrimination ability was compared using C‐indices. Consistency between the nomogram‐estimated 3‐ and 5‐year OS probabilities and actual OS probabilities was examined using a calibration plot, with the 45° line as the reference. Comparisons between the nomogram model and the single conventional predictors at 3 and 5 years were constructed using ROC analysis. Decision curve analysis (DCA) was used to assess the clinical utility and benefits of the prediction model. Ultimately, patients were divided into two risk groups based on median nomogram scores. We employed the inverse probability of treatment weighting (IPTW) to ensure a balanced comparison of baseline characteristics among patients receiving different treatment modalities in both the low‐ and high‐risk groups [30]. The non‐parametric test compared categorical variables between risk groups. All tests were two‐tailed, with significance set at *p* < 0.05.

## 3 Results

### 3.1 Patient Characteristics

Through retrospective selection, 308 patients treated from 2007 to 2019 were included. The baseline characteristics and treatment methods are presented in Table 1. Briefly, the age of the patients ranged from 11 to 76 years, with a median age of 46, and 81.5% were male. Of the 308 patients, 28.6% presented with one metastatic lesion. Osteoblastic bone metastasis was the predominant type, observed in 64.0% of cases. There were 148 (48.05%) and 160 (51.95%) patients presenting with pre‐treatment EBV DNA < and ≥ 10,000 copies/mL, respectively, and the median number of EBV DNA was 11,250 copies/mL of the whole cohort. All patients underwent tumor response assessment after PCT, and 224 (72.73%) achieved clinical partial response (PR) or complete response (CR) after PCT. The majority of bone metastatic lesions (36.36%) were found in the spinal zone, followed by regions involving multiple zones or extremities (33.77%), pelvic zone (17.21%), and thoracic zone (12.66%) sequentially.

**TABLE 1:** Patient characteristics in the cohort.

Characteristics	Overall (*n* = 308)	PCT alone (*n* = 69)	PCT + LRRT (*n* = 105)	PCT + LRRT + bRT (*n* = 134)	*p*
Age (years)					
≤ 45	145 (47.08)	30 (43.48)	49 (46.67)	66 (49.25)	0.733
> 45	163 (52.92)	39 (56.52)	56 (53.33)	68 (50.75)	
Sex					
Male	251 (81.49)	58 (84.06)	92 (87.62)	101 (75.37)	0.044
Female	57 (18.51)	11 (15.94)	13 (12.38)	33 (24.63)	
T stage					
1–2	49 (15.91)	8 (11.59)	20 (19.05)	21 (15.67)	0.419
3–4	259 (84.09)	61 (88.41)	85 (80.95)	113 (84.33)	
N stage					
0–1	56 (18.18)	15 (21.74)	20 (19.05)	21 (15.67)	0.547
2–3	252 (81.82)	54 (78.26)	85 (80.95)	113 (84.33)	
EBV DNA (copies/mL)					
< 10,000	148 (48.05)	31 (44.93)	44 (41.90)	73 (54.48)	0.130
≥ 10,000	160 (51.95)	38 (55.07)	61 (58.10)	61 (45.52)	
ALP (U/L)					
< 110	278 (90.26)	62 (89.86)	93 (88.57)	123 (91.79)	0.701
≥ 110	30 (9.74)	7 (10.14)	12 (11.43)	11 (8.21)	
LDH (U/L)					
< 250	262 (85.06)	52 (75.36)	90 (85.71)	120 (89.55)	0.026
≥ 250	46 (14.94)	17 (24.64)	15 (14.29)	14 (10.45)	
No. of metastatic lesions					
1–3	267 (86.69)	56 (81.16)	82 (78.10)	129 (96.27)	0.001
4–5	41 (13.31)	13 (18.84)	23 (21.90)	5 (3.73)	
Property					
Osteogenesis	197 (63.96)	37 (53.62)	66 (62.86)	94 (70.15)	0.065
Osteolysis	111 (36.04)	32 (46.38)	39 (37.14)	40 (29.85)	
PCT regimen					
TPF	93 (30.19)	9 (13.04)	32 (30.48)	52 (38.81)	0.001
TP	76 (24.68)	15 (21.74)	23 (21.90)	38 (28.36)	
PF	90 (29.22)	27 (39.13)	34 (32.38)	29 (21.64)	
GP	24 (7.79)	11 (15.94)	6 (5.71)	7 (5.22)	
Multiple or other regimes	25 (8.12)	7 (10.14)	10 (9.52)	8 (5.97)	
Response after PCT					
PR	224 (72.73)	48 (69.57)	78 (74.29)	98 (73.13)	0.784
PD/SD	84 (27.27)	21 (30.43)	27 (25.71)	36 (26.87)	
Administration of CCT					
Yes	125 (40.58)	0 (0.00)	52 (49.52)	73 (54.48)	< 0.001
No	183 (59.42)	69 (100.00)	53 (50.48)	61 (45.52)	
Any bone symptoms					
Yes	14 (4.54)	5 (7.25)	2 (1.90)	7 (5.22)	0.224
No	294 (95.46)	64 (92.75)	103 (98.10)	127 (94.78)	
Immunotherapy					
Yes	12 (3.90)	5 (7.25)	2 (1.90)	5 (3.73)	0.203
No	296 (96.10)	64 (92.75)	103 (98.10)	129 (96.27)	

Abbreviations: ALP, alkaline phosphatase; bRT, radiotherapy to metastatic bone; CCT, concurrent chemotherapy; CR, complete response; EBV, Epstein–Barr virus; GP, gemcitabine plus cisplatin; LDH, lactic dehydrogenase; LRRT, locoregional radiotherapy; PCT, palliative chemotherapy; PD progression disease; PF, cisplatin plus 5‐fluorouracil; PR, partial response; RT, radiotherapy; SD, stable disease; TP, paclitaxel plus cisplatin; TPF, paclitaxel plus cisplatin and 5‐fluorouracil.

Fourteen of 308 patients suffered from pain from metastatic bone lesions at the time of the initial diagnosis. These symptoms could be controlled by osteoprotective and analgesic medications, thus allowing patients to successfully complete PCT.

Of the cohort, 105 (34.09%) underwent LRRT, 134 (43.51%) had both LRRT and bRT on all metastatic bone lesions, while the rest had PCT only. Moreover, of the 142 patients who received bRT on at least one metastatic bone lesion, 67 (47.18%) patients received a radical dose of 60–70 Gy/28–33F, and the remaining patients were treated with bRT for palliative care. Among 224 patients who showed PR after PCT, 98 (43.75%) received both LRRT and bRT, 78 had LRRT, and 48 had PCT alone. CCT was given to 52 of 105 (49.52%) and 73 of 134 (54.48%) in the LRRT and LRRT+bRT groups, respectively.

### 3.2 Survival

As of December 5, 2020, the median follow‐up period was 37.2 months (IQR 21.3–74.9). A total of 176 patients experienced disease progression and 81 deaths occurred. The median PFS was 25.6 months (IQR 12–136.2) in the entire cohort. Among all progressive events, 48 progressed to other bones, and 99 patients developed metastasis to other organs, distant lymph nodes, or recurrence at the primary sites. The detailed progression information is shown in Table S1. After disease progression, 42 patients received best support care and 6 patients participated in clinical trial. Ninety patients underwent the subsequent‐line palliative chemotherapy, with or without immunotherapy and targeted therapy. And there were 38 patients received second‐line chemotherapy combined with local treatment for recurrence and metastasis, including radiotherapy, surgery, and ablation therapy. The 3‐year and 5‐year PFS rates of the entire cohort were 42.3% and 33.6%, respectively, while the 3‐year and 5‐year OS rates were 78.0% and 61.4%, respectively.

**FIGURE 2:**
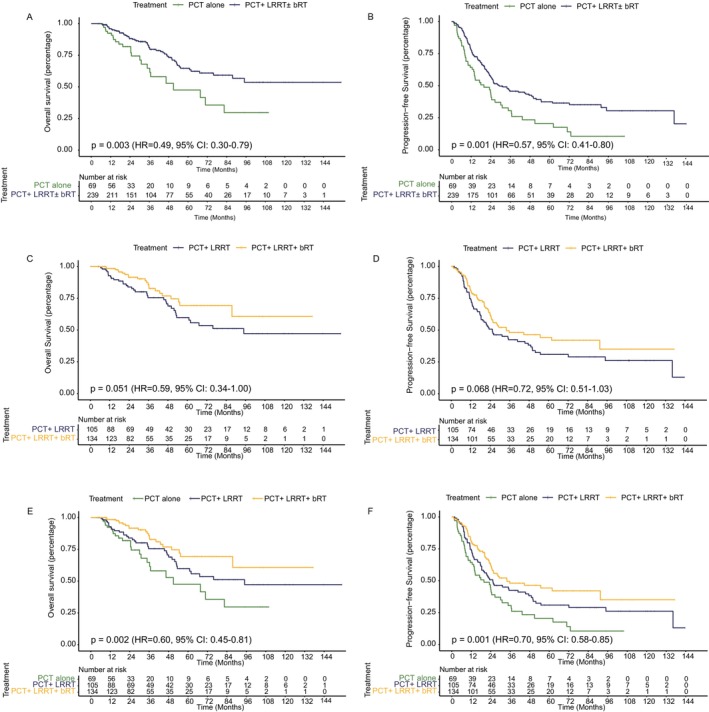
Comparison of overall survival and progression‐free survival in the different radiotherapy modalities groups. (A) Overall survival, (B) progression‐free survival comparing those who received PCT versus PCT + LRRT. (C) Overall survival, (D) progression‐free survival between patients receiving PCT + LRRT versus PCT + LRRT + bRT. (E) Overall survival, (F) progression‐free survival among groups receiving PCT alone, PCT + LRRT, and PCT + LRRT + bRT. PCT, palliative chemotherapy; LRRT, locoregional radiotherapy.

The 3‐year OS rates of patients who received PCT alone, PCT + LRRT, and PCT + LRRT + bRT were 64.7%, 75.5%, and 85.9%, respectively, and the 5‐year OS rates were 47.5%, 59.9%, and 69.4%, respectively. As shown in Figure 2A,B, patients who received LRRT with or without bRT had statistically superior OS and PFS (OS: HR = 0.49, 95% CI: 0.30–0.79, *p* = 0.003; PFS: HR = 0.57, 95% CI: 0.41–0.80, *p* = 0.001) compared to patients who received PCT alone, but the addition of bRT to LRRT improved the OS and PFS compared to that of patients who received PCT + LRRT only with borderline significance (OS: HR = 0.59, 95% CI: 0.34–1.00, *p* = 0.051; PFS: HR = 0.72, 95% CI: 0.51–1.03, *p* = 0.068; Figure 2C,D). Altogether, the best OS and PFS was observed in patients who received LRRT+bRT (OS: HR = 0.60, 95% CI: 0.45–0.81, *p* = 0.002; PFS: HR = 0.70, 95% CI: 0.58–0.85, *p* = 0.001; Figure 2E,F). The 3‐year PFS rates in the PCT alone, PCT + LRRT, and PCT + LRRT + bRT groups were 30.5%, 42.4%, and 48.1%, respectively, and the 5‐year PFS were 20.5%, 30.9%, and 44.3%, respectively.

### 3.3 Univariable Analysis and Multivariable Analysis

Table 2 shows clinical characteristics' impact on prognosis. Univariate analysis found older age (*p* = 0.034), advanced N stage (*p* = 0.007), 4–5 metastatic lesions (*p* = 0.046), high LDH (*p* < 0.001), and high EBV DNA (*p* < 0.001) negatively impacted OS. No significant OS association was found with sex, T stage, pre‐treatment ALP level, properties of metastatic lesions, PCT regimens, and response after PCT. In multivariate analysis, independent prognostic factors were age (HR = 1.66, 95% CI: 1.03–2.66, *p* = 0.036), N stage (HR = 2.2, 95% CI: 1.11–4.36, *p* = 0.024), LDH (HR = 1.97, 95% CI: 1.14–3.42, *p* = 0.016), and EBV DNA (HR = 2.34, 95% CI: 1.38–3.95, *p* = 0.002). Additionally, PCT + LRRT + bRT was an independent positive factor for OS (HR = 0.4, 95% CI: 0.22–0.74, *p* = 0.003).

**TABLE 2:** Univariable and multivariable analyses for OS.

Characteristics	Univariable analysis		Multivariable analysis	
	Hazard ratio (95% CI)	*p*	Hazard ratio (95% CI)	*p*
Treatment				
PCT alone	Reference		Reference	
PCT + LRRT	0.62 (0.37–1.06)	0.079	0.62 (0.36–1.07)	0.085
PCT + LRRT + bRT	0.37 (0.20–0.65)	0.001	0.4 (0.22–0.74)	**0.003**
Age (years)				
≤ 45	Reference		Reference	
> 45	1.64 (1.04–2.59)	0.034	1.66 (1.03–2.66)	**0.036**
Sex				
Male	Reference			
Female	0.67 (0.36–1.23)	0.194		
T stage				
1–2	Reference			
3–4	1.68 (0.86–3.26)	0.127		
N stage				
0–1	Reference		Reference	
2–3	2.5 (1.28–4.88)	0.007	2.2 (1.11–4.36)	**0.024**
EBV DNA level (copies/mL)				
< 10,000	Reference		Reference	
≥ 10,000	2.87 (1.73–4.76)	< 0.001	2.34 (1.38–3.95)	**0.002**
ALP (U/L)				
< 110	Reference			
≥ 110	1.67 (0.88–3.16)	0.116		
LDH (U/L)				
< 250	Reference		Reference	
≥ 250	2.7 (1.62–4.49)	< 0.001	1.97 (1.14–3.42)	**0.016**
No. of metastatic lesions				
1–3	Reference		Reference	
4–5	1.75 (1.01–3.02)	0.046	1.31 (0.74–2.32)	0.361
Property				
Osteogenesis	Reference			
Osteolysis	1.3 (0.84–2.02)	0.240		
PCT regimen				
TPF	Reference			
TP	1.29 (0.69–2.4)	0.420		
PF	1.36 (0.77–2.42)	0.293		
GP	0.9 (0.27–3.05)	0.872		
Multiple or other regimes	1.73 (0.81–3.7)	0.158		
Response after PCT				
PR	Reference			
PD/SD	1.3 (0.81–2.07)	0.278		
Administration of CCT				
No	Reference			
Yes	0.98 (0.63–1.53)	0.941		

*Note:* All analyses were conducted using the Cox regression model.

Furthermore, for patients with only 1 metastatic bone lesion, PCT+LRRT+bRT did not significantly improve OS (HR = 0.65, 95%CI: 0.44–0.96, *p* = 0.083) but improved PFS (HR = 0.68, 95%CI: 0.51–0.90, *p* = 0.019) compared to PCT alone or PCT+LRRT. We also investigated the impact of the addition of CCT to RT on survival in patients receiving LRRT± bRT. The 3‐ and 5‐year OS rates were 75.4% and 62% with CCT, and 87.8% and 68% without (HR = 1.40, 95% CI: 0.82–2.37, *p* = 0.211). We also noticed that patients with PR after PCT showed prolonged OS (HR = 0.46, 95% CI: 0.25–0.82, *p* = 0.008) and PFS (HR = 0.54, 95% CI: 0.36–0.80, *p* = 0.002) with LRRT. However, patients with stable or progressive disease after PCT failed to achieve better OS (HR = 0.59, 95% CI: 0.25–1.36, *p* = 0.209) and PFS (HR = 0.72, 95% CI: 0.40–1.29, *p* = 0.264) with the addition of LRRT.

### 3.4 Construction and Assessment of the Nomogram Model

**FIGURE 3:**
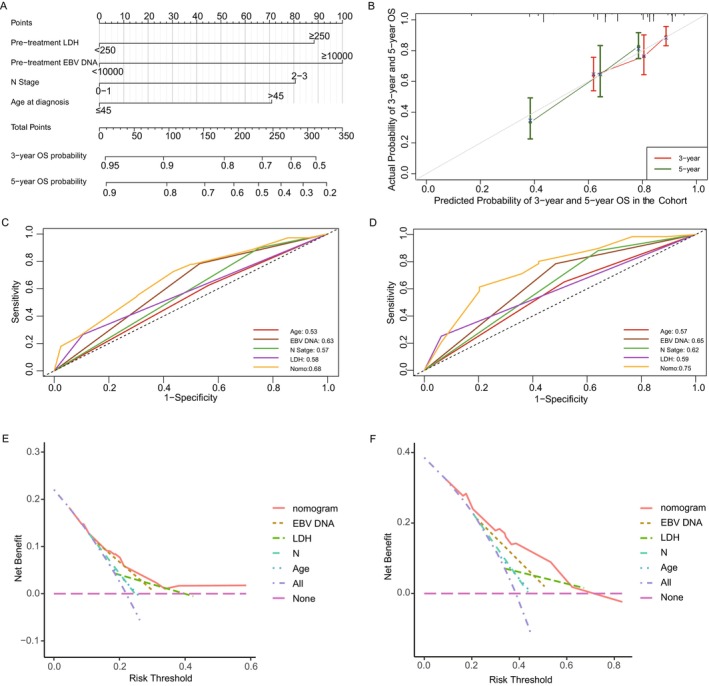
Construction and assessment of nomogram of the whole cohort. (A) Nomogram model predicting 3‐ and 5‐year OS in primary bone‐only oligometastatic NPC patients. (B) The calibration curves for predicting OS at 3 and 5 years. (C) ROC curves evaluating the nomogram against its four individual variables for 3‐year OS predictions. (D) ROC curves evaluating the nomogram against its four individual variables for 5‐year OS predictions. (E) DCA assessing the predictive utility of the nomogram and its four component variables for 3‐year OS. (F) DCA assessing the predictive utility of the nomogram and its four component variables for 5‐year OS. ROC, receiver operating characteristic; DCA, decision curve analysis.

A nomogram was developed incorporating independent prognostic factors to predict survival and classify risk. In this nomogram model, pre‐treatment EBV DNA level had the greatest effect on OS, followed by N stage, LDH level, and age at diagnosis (Figure 3A). Nomogram performance was evaluated graphically using a calibration plot adjusted by bootstrapping with 1000 samples (Figure 3B). Model performance was validated using a 1000‐sample bootstrapped calibration plot (Figure 3B), showing good overlap for 3‐ and 5‐year OS rates. The C‐index was 0.71 (95% CI: 0.64–0.78). The model outperformed single indicators in predictive power, as assessed by C‐index and ROC curve comparisons (Figure 3C,D).

DCA showed the nomogram had the highest net benefit for 3‐ and 5‐year OS rates compared to single predictors (Figure 3E,F), with top net benefits of 0.182 and 0.323, respectively. Furthermore, risk threshold probabilities varied from 4.7% to 58.6% for 3‐year and 9.2% to 83.3% for 5‐year OS. The nomogram yielded good net clinical benefit for predicting OS when the 3‐ and 5‐year OS threshold probabilities were below 58.6% and 62.3%, respectively.

### 3.5 Treatment Strategy for Different Risk Groups

**FIGURE 4:**
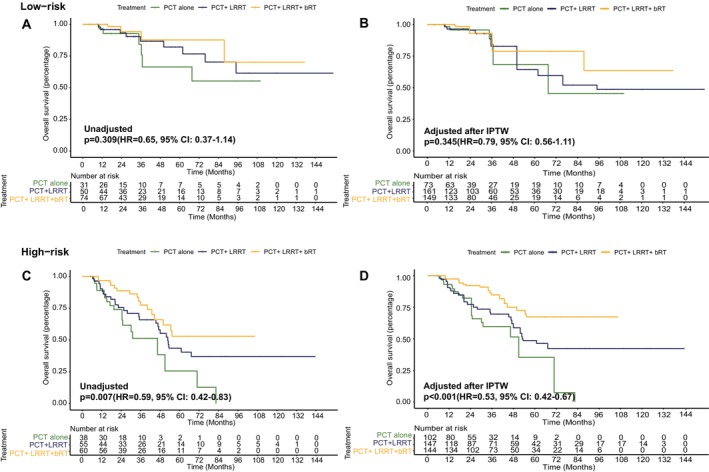
Comparison of overall survival between low‐risk and high‐risk groups based on the predictor from the nomogram model and impact of radiotherapy modalities in two groups. (A) Unadjusted Kaplan–Meier curves of OS for low‐risk patients receiving PCT alone, PCT + LRRT, and PCT + LRRT + bRT; (B) adjusted Kaplan–Meier curves of OS for low‐risk patients receiving PCT alone, PCT + LRRT, and PCT + LRRT + bRT; (C) unadjusted Kaplan–Meier curves of OS for high‐risk patients receiving PCT alone, PCT + LRRT, and PCT + LRRT + bRT; (D) adjusted Kaplan–Meier curves of OS for high‐risk patients receiving PCT alone, PCT + LRRT, and PCT + LRRT + bRT.

We scored 308 patients and divided them into low‐ and high‐risk groups (Figure 4A), with 155 (50.32%) in the low‐risk and 153 (49.68%) in the high‐risk group. High‐risk patients were older, had advanced N stage, and higher pre‐treatment EBV DNA and LDH levels. No significant differences in PCT response (*p* = 0.650) or treatment approaches (*p* = 0.301) existed between groups (Table S2). And after IPTW adjustment, the baseline characteristics among different treatment groups showed no significant differences in both low‐ and high‐risk groups (all *p* > 0.05, Tables S3 and S4).

The 3‐year and 5‐year OS of low‐ and high‐risk groups were 89.7% versus 67.5% and 81.9% versus 44.5%, respectively (median OS: not reached vs. 53.0 months, HR = 0.32, 95% CI: 0.19–0.52, *p* < 0.001; Figure S2A). The 3‐year and 5‐year PFS of the low‐risk group were 56.6% and 48.1%, and for the high‐risk group, they were 29.8% and 21.3% (median PFS: 50.1 months vs. 22.2 months, HR = 0.53, 95% CI: 0.39–0.72, *p* < 0.001; Figure S2B). Figure 4A shows that in the low‐risk group, there was no significant difference in OS among the three treatment modalities. Detailed results of the two‐by‐two comparisons are shown in Table S5 (3‐year OS: 84.3% vs. 86.7% vs. 94.2%, 5‐year OS: 66.4% vs. 82.1% vs. 87.7%, HR = 0.65, 95% CI: 0.37–1.14, *p* = 0.309). The adjusted Kaplan–Meier curves after IPTW also did not show a significant difference in OS among patients receiving PCT alone, PCT + LRRT, and PCT + LRRT + bRT (HR = 0.79, 95% CI: 0.56–1.11, *p* = 0.345; Figure 4B). On the other hand, in the high‐risk group, the adjusted Kaplan–Meier curves showed a significant improvement in OS for patients who underwent PCT + LRRT + bRT (HR = 0.53, 95% CI: 0.42–0.67, *p* < 0.001; Figure 4D), as did the unadjusted Kaplan–Meier curves (HR = 0.59, 95% CI: 0.42–0.83, *p* = 0.007; Figure 4C).

## 4 Discussion

Bone is the most affected organ in mNPC [6, 31]. Due to the limitations of the TNM staging system and the heterogeneity of mNPC, a more accurate risk assessment and tailored treatment approach are needed.The 3‐year and 5‐year OS rates of primary bone‐only oligometastases in our study were 78.0% and 61.4%, respectively, which were better than the survival rates of primary oligometastatic or bone‐only mNPC patients reported in previous studies [32, 33]. Therefore, treatment of bone‐only oligometastatic NPC should be tailored. However, only a few studies have focused on this population. Our study was the largest to date, focusing exclusively on this population. We identified key prognostic factors, such as age, pre‐treatment levels of EBV and LDH, and N stage, and stratified patients by risk levels. Our findings suggest that bRT significantly improved OS, especially for high‐risk patients.

There have been a few previous studies on primary bone‐only mNPC. Sun et al. [15] collected data of 226 primary bone‐only metastatic NPC patients and found that the EBV DNA level after PCT (undetectable vs. detectable) and number of metastatic lesions (≤ 3 vs. > 3) were independent prognostic factors and thus classified patients into high‐, intermediate‐, and low‐risk groups. However, their study differed from ours in several ways. Unlike us, the survival benefit from bRT was not observed in all risk groups in their study. This discrepancy could stem from the heterogeneity of their study population, which wasn't limited to oligometastatic patients, reflected by their low 3‐year and 5‐year OS compared to ours. Another influencing factor could be the small subset of their cohort (68/226) receiving bone radiotherapy, potentially skewing the assessment of its impact. Lin et al. [16] collected data of 131 patients with primary bone‐only metastatic NPC, of whom 88 (67.2%) patients were oligometastatic, and suggested that oligometastatic patients who received bRT tended to have a better OS (83.0 months vs. 45.0 months) and PFS (60 months vs. 36.5 months). However, their results were not statistically significant, which might be because of the small sample sizes as well.

Several previous studies have suggested that the addition of LRRT to PCT could further improve the survival of patients with primary metastatic NPC [19, 34], particularly in those that responded better to PCT [6, 21]. An important randomized clinical trial conducted by You et al. [21] investigated whether primary mNPC could benefit from LRRT. You et al. randomly assigned 126 primary metastatic NPC patients who achieved CR or PR after receiving three cycles of PCT to the LRRT group or observation group and found that patients in the LRRT group had significantly longer OS and PFS than those who received only PCT. Similar findings were obtained in our study, in that the addition of LRRT in patients who achieved PR after PCT resulted in longer OS and PFS.

Moreover, after risk stratification, we found that high‐risk patients benefited from PCT + LRRT + bRT, a more aggressive treatment strategy, whereas low‐risk patients did not. One reason could be that the low‐risk group, characterized by lower tumor burden and invasiveness, generally responded well to chemotherapy alone. In our study, the 3‐year OS in the low‐risk group was 89.7%, significantly better than that of metastatic NPC reported previously but were comparable to the 3‐year OS rates of 86.9%–94.7% reported for advanced stage NPC (Stage III–IVB) in other clinical trials [35, 36, 37, 38]. In the low‐risk group, 74.19% of patients responded well to PCT, indicating a general high sensitivity to PCT. These patients may have longer tumor‐free survival with just PCT, making the role of RT less clear for them. Further long‐term studies are needed to balance the potential survival gains against the risks of multi‐site radiation side effects [24].

For high‐risk patients with elevated EBV and LDH levels, PCT alone may not be sufficient due to a higher likelihood of progression [39, 40]. RT can completely eradicate BM with a radical dose [27] and also helps in palliative pain relief and the prevention of skeletal‐related events [41]. Additionally, RT may induce an immune‐mediated abscopal effect [42], which has been shown to increase survival in cancers of the lungs, liver [43, 44]. However, more aggressive treatments may not be suitable for everyone, like the elderly, due to potential side effects. Therefore, individual factors like life expectancy and physical condition should guide the use of bRT. When applicable, less invasive techniques like SBRT should be considered [45].

The integration of immunotherapy with PCT is becoming the standard approach for treating mNPC, a subject of intense current research. At the cellular level, local RT may trigger immunogenic cell death. This stimulates systemic inflammation and activates antigen‐presenting dendritic cells and cytotoxic T cells, thereby enhancing the body's anti‐cancer immune response [46]. Additionally, preclinical [47] and preliminary clinical evidence [48, 49] indicate that the local use of RT in conjunction with immunotherapy might trigger antigen release and T‐cell activation, boosting the local and systemic effects of immunotherapy. This has been supported by Phase II trials in various cancers, such as non‐small cell lung cancer [50], microsatellite stable colorectal and pancreatic adenocarcinoma [51]. Aside from solid tumors, in the realm of hematological malignancies, Al‐Ibraheem and his colleagues observed the abscopal effect of local RT in lymphoma patients who underwent radiotherapy combined with immunotherapy, as evidenced by PET‐CT scans [52]. However, the survival impact of adding local RT to immunotherapy in metastatic NPC is still unknown and needs further study.

This study has some limitations. It is retrospective, which may introduce selection bias. The data, from a single facility, might not represent all patients with primary bone‐only oligometastatic NPC across different regions. The benefits of local RT in the age of immunotherapy remain uncertain and warrant prospective trials. One significant limitation was the lower number of events in the low‐risk group, which may impact the statistical significance of the survival analysis. Although we used the IPTW method to address this issue, a larger sample size is needed in future studies to confirm our findings. In the end, although we found that high‐risk patients are more likely to benefit from an aggressive treatment pattern, and bRT does play a role in improving OS for high‐risk patients, further multicenter prospective studies are needed to compare the difference of efficacy between PCT + LRRT and PCT + LRRT + bRT.

## 5 Conclusion

In our study, different treatment regimens led to varying OS and PFS rates in primary bone‐only oligometastatic NPC patients. Best outcomes were seen with PCT + LRRT+bRT. Age, N stage, LDH, and EBV DNA were key prognostic factors. With the development of a nomogram model, we classified patients into high‐ and low‐risk groups based on their median prognostic scores and found that patients in the low‐risk group had a better prognosis but could not benefit from bRT, whereas patients in the high‐risk group achieve a longer OS from the PCT + LRRT+bRT regimen. The role of bRT in the era of immunotherapy needs further study.

## Author Contributions

**Wan‐Ping Guo:** data curation (equal), formal analysis (equal), investigation (equal), visualization (equal), writing – original draft (equal). **Guo‐Dong Jia:** data curation (equal), formal analysis (equal), investigation (equal), visualization (equal). **Si‐Yi Xie:** validation (equal). **Xuan Yu:** data curation (equal). **Xiao‐Han Meng:** validation (equal). **Lin‐Quan Tang:** methodology (equal), supervision (equal), writing – review and editing (equal). **Xiao‐Yun Li:** conceptualization (equal), project administration (equal), supervision (equal), writing – review and editing (equal). **Dong‐Hua Luo:** conceptualization (equal), project administration (equal), supervision (equal), writing – review and editing (equal).

## Ethics Statement

This study was approved by the Human Ethics Approval Committee of Sun Yat‐sen University Cancer Center (No. B2023‐575‐01) and informed consents were obtained from all patients.

## Conflicts of Interest

The authors declare no conflicts of interest.

## Supporting information


**Figure S1.** Comparison of two ROC curves for EBV DNA cutoff.

**Figure S2.** Comparison of overall survival and progression‐free survival in the low‐ and high‐risk groups. (A) Overall survival and (B) progression‐free survival.

**Table S1.** Detailed progression pattern and PFS results.
**Table S2.** Patient characteristics in low‐risk and high‐risk groups.
**Table S3.** Baseline characteristics of low‐risk patients receiving different treatment in unadjusted and IPTW‐adjusted study.
**Table S4.** Baseline characteristics of high‐risk patients receiving different treatment in unadjusted and IPTW‐adjusted study populations.
**Table S5.** Detailed results among three treatment modalities in two risk groups.

## Data Availability

Due to the nature of this research, participants of this study did not agree for their data to be shared publicly, so supporting data are not available.
