# Supplementary figures and images for: Whether Primary Bone‐Only Oligometastatic Nasopharyngeal Carcinoma Patients Benefit From Radiotherapy to the Bones on the Basis of Palliative Chemotherapy Plus Locoregional Radiotherapy?—A Large‐Cohort Retrospective Study
Source: Cancer Med. 2024 Nov 4;13(21):e70315. doi: 10.1002/cam4.70315 (PMC11533001; doi:10.1002/cam4.70315)

Comparison of two ROC curves for EBV DNA cutoff

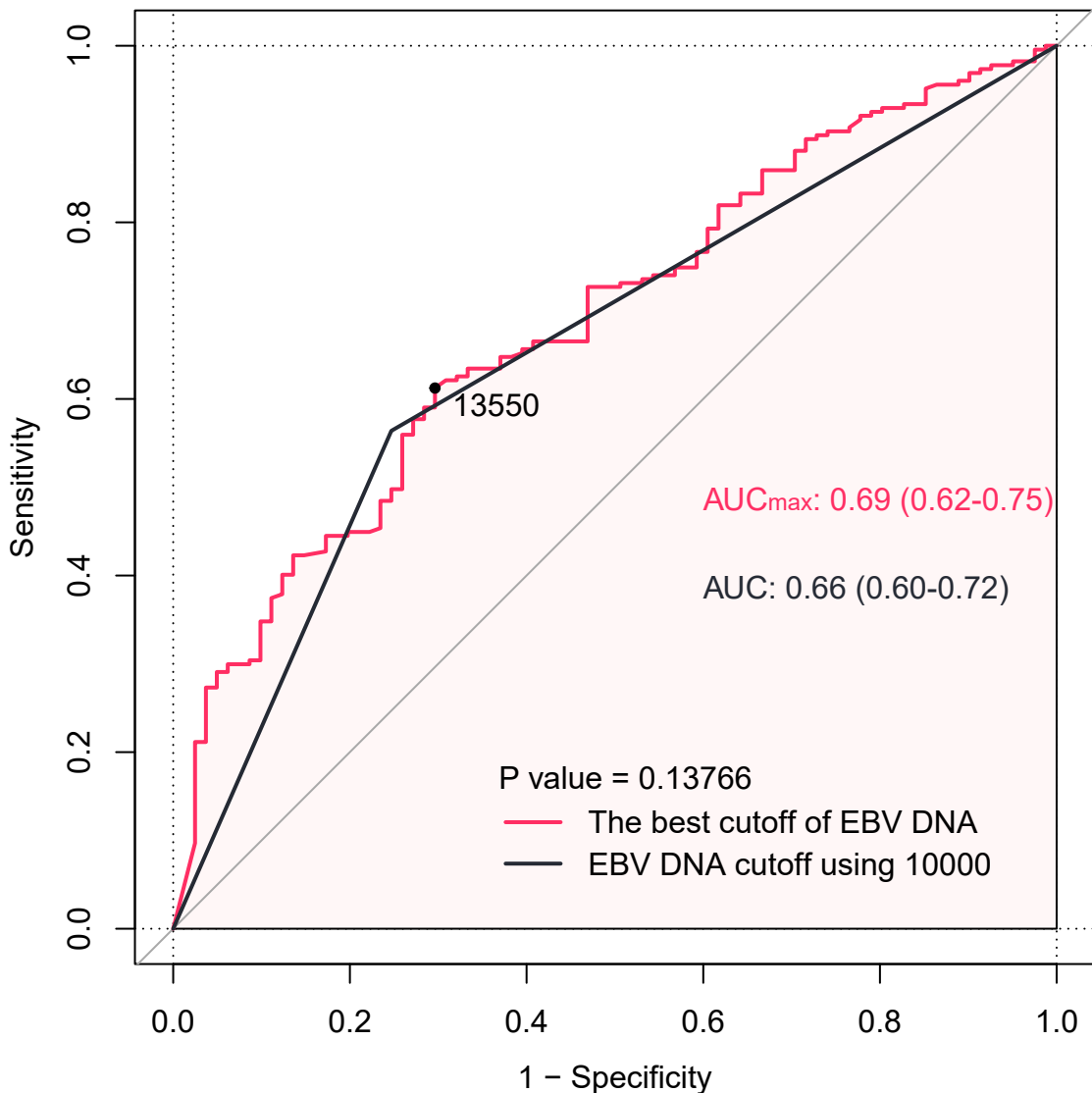

Supplement: Supplementary file 1 — Figure S1. Comparison of two ROC curves for EBV DNA cutoff. [file CAM4-13-e70315-s001.pdf]

Groups — Low-risk group — High-risk group

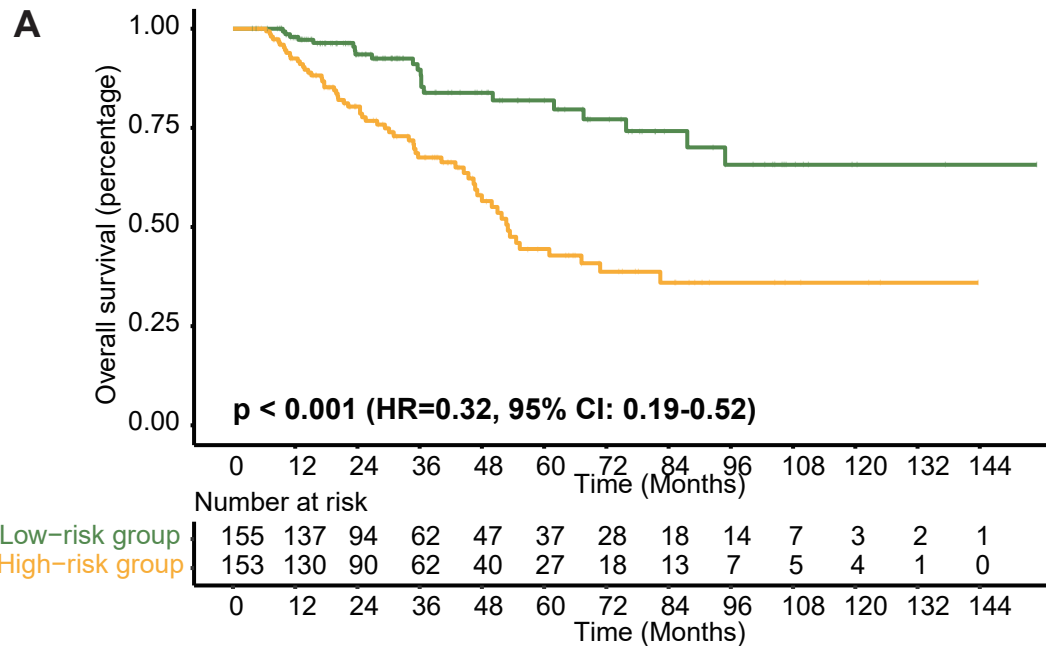

Groups — Low-risk group — High-risk group

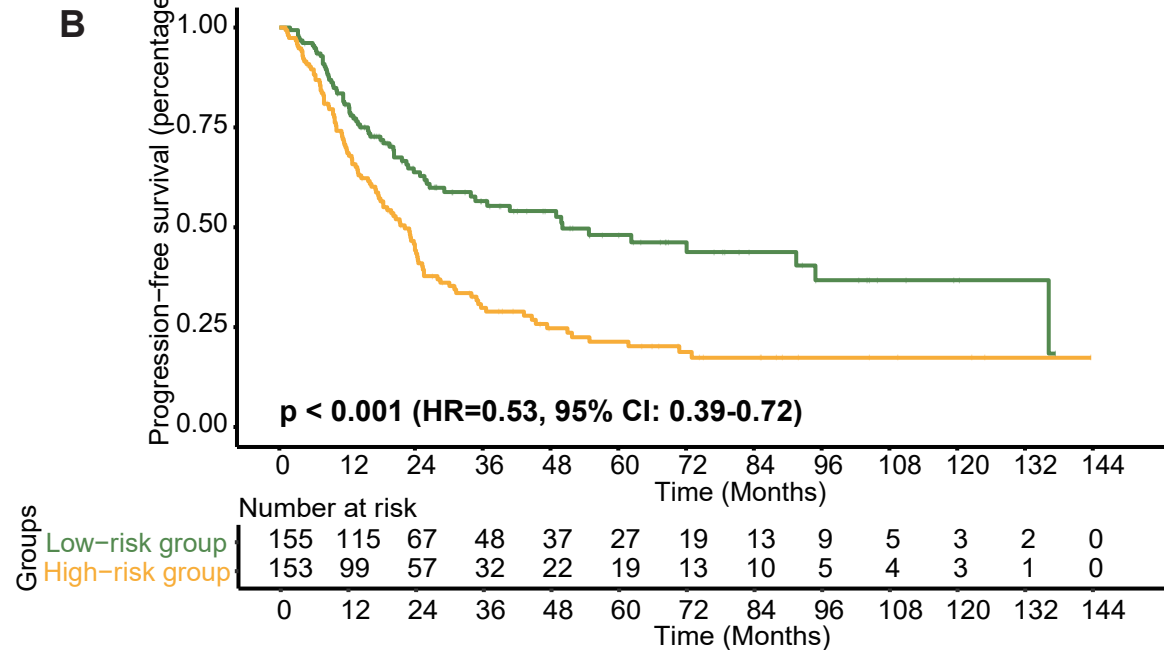

Supplement: Supplementary file 2 — Figure S2. Comparison of overall survival and progression‐free survival in the low‐ and high‐risk groups. (A) Overall survival and (B) progression‐free survival. [file CAM4-13-e70315-s003.pdf]
