# Supplementary material for: Whether Primary Bone‐Only Oligometastatic Nasopharyngeal Carcinoma Patients Benefit From Radiotherapy to the Bones on the Basis of Palliative Chemotherapy Plus Locoregional Radiotherapy?—A Large‐Cohort Retrospective Study
Source: Cancer Med. 2024 Nov 4;13(21):e70315. doi: 10.1002/cam4.70315 (PMC11533001; doi:10.1002/cam4.70315)
Supplement: Supplementary file 3 — Table S1. Detailed progression pattern and PFS results. Table S2. Patient characteristics in low‐risk and high‐risk groups. Table S3. Baseline characteristics of low‐risk patients receiving different treatment in unadjusted and IPTW‐adjusted study. Table S4. Baseline characteristics of high‐risk patients receiving different treatment in unadjusted and IPTW‐adjusted study populations. Table S5. Detailed results among three treatment modalities in two risk groups. [file CAM4-13-e70315-s002.docx]

**Table S1** Detailed progression pattern and PFS results

|  | Overall  (n=308) | PCT alone  (n=69) | PCT + LRRT  (n=105) | PCT + LRRT + bone RT  (n=134) |
| --- | --- | --- | --- | --- |
| **Progression Pattern** |  | | | |
| Bone metastasis only | 48 (15.6%) | 7 (10.1%) | 18 (17.1%) | 23 (17.2%) |
| Single organ (liver/lung/distant lymph node) metastasis | 47 (15.3%) | 7 (10.1%) | 19 (18.1%) | 21 (15.7%) |
| Nasopharyngeal or cervical lymph node recurrent only | 17 (5.5%) | 10 (14.5%) | 5 (4.8%) | 2 (1.5%) |
| Multi-metastasis and recurrent | 79 (25.6%) | 18 (5.8%) | 27 (25.7%) | 34 (25.4%) |
| Progression during treatment | 20 (6.5%) | 10 (14.5%) | 5 (4.8%) | 5 (3.7%) |
| **PFS rates** |  |  |  |  |
| 3-year PFS | 42.3% | 30.5% | 42.4% | 48.1% |
| 5-year PFS | 33.6% | 20.5% | 30.9% | 44.3% |

**Table S2** Patient characteristics in low-risk and high-risk groups.

| Characteristics | Low-risk Group | High-risk Group | P value |
| --- | --- | --- | --- |
| **Overall** | 155 (50.32%) | 153 (49.68%) |  |
| **Age (years)** |  |  | 0.030 |
| ≤ 45 | 83 (53.55) | 62 (40.52) |  |
| > 45 | 72 (46.45) | 91 (59.48) |  |
| **Sex** |  |  | 0.957 |
| Male | 127 (81.94) | 124 (81.05) |  |
| Female | 28 (18.06) | 29 (18.95) |  |
| **T Stage** |  |  | 0.376 |
| 1-2 | 28 (18.06) | 21 (13.73) |  |
| 3-4 | 127 (81.94) | 132 (86.27) |  |
| **N Stage** |  |  | 0.002 |
| 0-1 | 39 (25.16) | 17 (11.11) |  |
| 2-3 | 116 (74.84) | 136 (88.89) |  |
| **EBV DNA (copies/mL)** |  |  | <0.001 |
| < 10,000 | 146 (94.19) | 2 (1.31) |  |
| ≥ 10,000 | 9 (5.81) | 151 (98.69) |  |
| **ALP (U/L)** |  |  | 1 |
| < 110 | 140 (90.32) | 138 (90.20) |  |
| ≥ 110 | 15 (9.68) | 15 (9.80) |  |
| **LDH (U/L)** |  |  | <0.001 |
| < 250 | 146 (94.19) | 116 (75.82) |  |
| ≥ 250 | 9 (5.81) | 37 (24.18) |  |
| **No. of Metastatic lesions** |  |  | 0.964 |
| 1-3 | 135 (87.10) | 132 (86.27) |  |
| 4-5 | 20 (12.90) | 21 (13.73) |  |
| **Property** |  |  | 0.014 |
| Osteogenesis | 110 (70.97) | 87 (56.86) |  |
| Osteolysis | 45 (29.03) | 66 (43.14) |  |
| **PCT Regimen** |  |  | 0.672 |
| TPF | 49 (31.61) | 44 (28.76) |  |
| TP | 37 (23.87) | 39 (25.49) |  |
| PF | 43 (27.74) | 47 (30.72) |  |
| GP | 15 (9.68) | 9 (5.88) |  |
| Multiple or Other Regimes | 11 (7.10) | 14 (9.15) |  |
| **Response after PCT** |  |  | 0.650 |
| PR | 115 (74.19) | 109 (71.24) |  |
| PD/SD | 40 (25.81) | 44 (28.76) |  |
| **Administration of CCT** |  |  | 0.078 |
| Yes | 71 (45.81) | 54 (35.29) |  |
| No | 84 (54.19) | 99 (64.71) |  |
| **Treatment modality**  PCT alone | 31 (20.00) | 38 (24.84) | 0.302 |
| PCT+ LRRT  PCT+ LRRT+ bone RT  **OS**  Median OS  3-year  5-year  **PFS**  Median PFS  3-year  5-year | 50 (32.26)  74 (47.74)  Not reached  89.7%  81.9%  50.1 months  56.6%  48.1% | 55 (35.95)  60 (39.22)  53.0 months  67.5%  44.5%  22.2 months  29.8%  21.3% | <0.001  (HR=0.32,  95%CI:0.19-0.52)  <0.001  (HR=0.53,  95%CI: 0.39-0.72) |

Note: Comparisons between two groups were conducted using the non-parametric test. 3-year and 5-year OS and PFS between two groups were calculated using the Kaplan-Meier survival curves and log-rank test.

**Table S3** Baseline characteristics of low-risk patients receiving different treatment in unadjusted and IPTW-adjusted study populations

|  | Overall cohort | | | | | IPTW-adjusted cohort | | | | |
| --- | --- | --- | --- | --- | --- | --- | --- | --- | --- | --- |
| Characteristics | Overall | PCT  alone | PCT  +LRRT | PCT+LRRT+ bRT | P value | Overall | PCT alone | PCT+  LRRT | PCT+LRRT  + bRT | P value |
| **Overall** | 155 | 31 | 50 | 74 |  | 382.41 | 73.12 | 160.54 | 148.74 |  |
| **Age (years)** |  |  |  |  | 0.456 |  |  |  |  | 0.591 |
| ≤ 45 | 83  (53.55) | 17 (54.84) | 30 (60.00) | 36  (48.65) |  | 199.41 (52.15) | 34.19 (46.75) | 93.45 (58.21) | 71.77 (48.25) |  |
| > 45 | 72 (46.45) | 14 (45.16) | 20 (40.00) | 38  (51.35) |  | 183.00 (47.85) | 38.94 (53.25) | 67.09 (41.79) | 76.97 (51.75) |  |
| **Sex** |  |  |  |  | < 0.001 |  |  |  |  | 0.646 |
| Male | 127 (81.94) | 29 (93.55) | 47 (94.00) | 51  (68.92) |  | 307.83 (80.50) | 64.77 (88.57) | 122.65 (76.40) | 120.41 (80.95) |  |
| Female | 28 (18.06) | 2  (6.45) | 3  (6.00) | 23  (31.08) |  | 74.57 (19.50) | 8.36 (11.43) | 37.89 (23.60) | 28.33 (19.05) |  |
| **T Stage** |  |  |  |  | 0.680 |  |  |  |  | 0.812 |
| 1-2 | 28 (18.06) | 5  (16.13) | 11 (22.00) | 12  (16.22) |  | 58.86 (15.39) | 13.44 (18.38) | 26.11 (16.26) | 19.31 (12.98) |  |
| 3-4 | 127 (81.94) | 26 (83.87) | 39 (78.00) | 62  (83.78) |  | 323.55 (84.61) | 59.69 (81.62) | 134.43 (83.74) | 129.43 (87.02) |  |
| **N Stage** |  |  |  |  | 0.164 |  |  |  |  | 0.830 |
| 0-1 | 39 (25.16) | 8  (25.81) | 17 (34.00) | 14  (18.92) |  | 93.00 (24.32) | 15.21 (20.79) | 37.18 (23.16) | 40.61 (27.30) |  |
| 2-3 | 116 (74.84) | 23 (74.19) | 33 (66.00) | 60  (81.08) |  | 289.41 (75.68) | 57.92 (79.21) | 123.36 (76.84) | 108.14 (72.70) |  |
| **EBV DNA (copies/mL)** |  |  |  |  | 0.009 |  |  |  |  | 0.467 |
| < 10,000 | 146 (94.19) | 31 (100.00) | 43 (86.00) | 72  (97.30) |  | 365.73 (95.64) | 73.12(100.00) | 151.52 (94.38) | 141.09 (94.85) |  |
| ≥ 10,000 | 9  (5.81) | 0  (0.00) | 7  (14.00) | 2  (2.70) |  | 16.67 (4.36) | 0.00 (0.00) | 9.02 (5.62) | 7.65  (5.15) |  |
| **ALP (U/L)** |  |  |  |  | 0.497 |  |  |  |  | 0.831 |
| < 110 | 140 (90.32) | 27 (87.10) | 44 (88.00) | 69  (93.24) |  | 351.57 (91.94) | 67.99 (92.98) | 145.26 (90.48) | 138.32 (92.99) |  |
| ≥ 110 | 15  (9.68) | 4  (12.90) | 6  (12.00) | 5  (6.76) |  | 30.84 (8.06) | 5.13 (7.02) | 15.28 (9.52) | 10.42  (7.01) |  |
| **LDH (U/L)** |  |  |  |  | 0.050 |  |  |  |  | 0.648 |
| < 250 | 146 (94.19) | 27 (87.10) | 46 (92.00) | 73  (98.65) |  | 356.05 (93.11) | 66.81 (91.37) | 153.85 (95.83) | 135.39 (91.02) |  |
| ≥ 250 | 9  (5.81) | 4  (12.90) | 4  (8.00) | 1  (1.35) |  | 26.36 (6.89) | 6.31 (8.63) | 6.69 (4.17) | 13.36  (8.98) |  |
| **No. of Metastatic lesions** |  |  |  |  | 0.007 |  |  |  |  | 0.506 |
| 1-3 | 135 (87.10) | 25 (80.65) | 39 (78.00) | 71  (95.95) |  | 342.36 (89.53) | 61.79 (84.50) | 141.97 (88.43) | 138.60 (93.18) |  |
| 4-5 | 20 (12.90) | 6  (19.35) | 11 (22.00) | 3  (4.05) |  | 40.05 (10.47) | 11.33 (15.50) | 18.57 (11.57) | 10.14  (6.82) |  |
| **Property** |  |  |  |  | 0.096 |  |  |  |  | 0.326 |
| Osteogenesis | 110 (70.97) | 18 (58.06) | 34 (68.00) | 58  (78.38) |  | 278.95 (72.95) | 44.22 (60.47) | 119.24 (74.27) | 115.49 (77.64) |  |
| Osteolysis | 45 (29.03) | 13 (41.94) | 16 (32.00) | 16  (21.62) |  | 103.46 (27.05) | 28.90 (39.53) | 41.30 (25.73) | 33.25 (22.36) |  |
| **PCT Regimen** |  |  |  |  | 0.006 |  |  |  |  | 0.476 |
| TPF | 49 (31.61) | 4  (12.90) | 13 (26.00) | 32  (43.24) |  | 101.77 (26.61) | 10.27 (14.05) | 39.99 (24.91) | 51.51 (34.63) |  |
| TP | 37 (23.87) | 6  (19.35) | 12 (24.00) | 19  (25.68) |  | 103.60 (27.09) | 17.71 (24.22) | 52.62 (32.78) | 33.27 (22.37) |  |
| PF | 43 (27.74) | 13 (41.94) | 16 (32.00) | 14  (18.92) |  | 117.36 (30.69) | 29.04 (39.71) | 49.29 (30.70) | 39.03 (26.24) |  |
| GP | 15  (9.68) | 7  (22.58) | 3  (6.00) | 5  (6.76) |  | 36.96 (9.66) | 14.51 (19.84) | 8.51 (5.30) | 13.94  (9.37) |  |
| Multiple or other regimes | 11  (7.10) | 1  (3.23) | 6  (12.00) | 4 (5.41) |  | 22.72 (5.94) | 1.59 (2.17) | 10.14 (6.32) | 10.99  (7.39) |  |
| **Response after PCT** |  |  |  |  | 0.385 |  |  |  |  | 0.849 |
| PR | 115 (74.19) | 20 (64.52) | 38 (76.00) | 57  (77.03) |  | 287.19 (75.10) | 51.48 (70.40) | 121.75 (75.84) | 113.96 (76.61) |  |
| PD/SD | 40 (25.81) | 11 (35.48) | 12 (24.00) | 17  (22.97) |  | 95.22 (24.90) | 21.65 (29.60) | 38.79 (24.16) | 34.79 (23.39) |  |
| **Administration of CCT** |  |  |  |  | NA |  |  |  |  | NA |
| No | 84 (54.19) | 31 (100.00) | 21 (42.00) | 32  (43.24) |  | 236.66 (61.89) | 73.12(100.00) | 85.53 (53.28) | 78.00 (52.44) |  |
| Yes | 71 (45.81) | 0  (0.00) | 29 (58.00) | 42  (56.76) |  | 145.75 (38.11) | 0.00 (0.00) | 75.01 (46.72) | 70.74 (47.56) |  |

**Table S4** Baseline characteristics of high-risk patients receiving different treatment in unadjusted and IPTW-adjusted study populations

|  | Overall cohort | | | | | IPTW-adjusted cohort | | | | |
| --- | --- | --- | --- | --- | --- | --- | --- | --- | --- | --- |
| Characteristics | Overall | PCT  alone | PCT  +LRRT | PCT+LRRT+ bRT | P value | Overall | PCT alone | PCT+  LRRT | PCT+LRRT  + bRT | P value |
| Overall | 153 | 38 | 55 | 60 |  | 392.64 | 101.65 | 146.82 | 144.17 |  |
| Age (years) |  |  |  |  | 0.159 |  |  |  |  | 0.644 |
| ≤ 45 | 62 (40.52) | 13 (34.21) | 19 (34.55) | 30  (50.00) |  | 153.57 (39.11) | 32.73 (32.20) | 58.91 (40.13) | 61.93 (42.95) |  |
| > 45 | 91 (59.48) | 25 (65.79) | 36 (65.45) | 30  (50.00) |  | 239.07 (60.89) | 68.92 (67.80) | 87.91 (59.87) | 82.24 (57.05) |  |
| Sex |  |  |  |  | 0.667 |  |  |  |  | 0.914 |
| Male | 124 (81.05) | 29 (76.32) | 45 (81.82) | 50  (83.33) |  | 321.78 (81.95) | 84.62 (83.25) | 121.64 (82.85) | 115.52 (80.13) |  |
| Female | 29 (18.95) | 9  (23.68) | 10 (18.18) | 10  (16.67) |  | 70.86 (18.05) | 17.03 (16.75) | 25.18 (17.15) | 28.65 (19.87) |  |
| T Stage |  |  |  |  | 0.473 |  |  |  |  | 0.960 |
| 1-2 | 21 (13.73) | 3  (7.89) | 9  (16.36) | 9  (15.00) |  | 57.68 (14.69) | 16.32 (16.06) | 21.60 (14.71) | 19.76 (13.70) |  |
| 3-4 | 132 (86.27) | 35 (92.11) | 46 (83.64) | 51  (85.00) |  | 334.96 (85.31) | 85.33 (83.94) | 125.22 (85.29) | 124.41 (86.30) |  |
| N Stage |  |  |  |  | 0.145 |  |  |  |  | 0.474 |
| 0-1 | 17 (11.11) | 7  (18.42) | 3  (5.45) | 7  (11.67) |  | 41.13 (10.48) | 16.37 (16.11) | 12.78 (8.70) | 11.98  (8.31) |  |
| 2-3 | 136 (88.89) | 31 (81.58) | 52 (94.55) | 53  (88.33) |  | 351.50 (89.52) | 85.28 (83.89) | 134.04 (91.30) | 132.19 (91.69) |  |
| EBV DNA (copies/mL) |  |  |  |  | 0.714 |  |  |  |  | 0.708 |
| < 10,000 | 2  (1.31) | 0  (0.00) | 1  (1.82) | 1  (1.67) |  | 3.96 (1.01) | 0.00 (0.00) | 1.98 (1.35) | 1.98  (1.37) |  |
| ≥ 10,000 | 151 (98.69) | 38 (100.00) | 54 (98.18) | 59  (98.33) |  | 388.68 (98.99) | 101.65(100.0) | 144.84 (98.65) | 142.19 (98.63) |  |
| ALP (U/L) |  |  |  |  | 0.889 |  |  |  |  | 0.983 |
| < 110 | 138 (90.20) | 35 (92.11) | 49 (89.09) | 54  (90.00) |  | 353.15 (89.94) | 90.60 (89.13) | 132.58 (90.30) | 129.96 (90.15) |  |
| ≥ 110 | 15  (9.80) | 3  (7.89) | 6  (10.91) | 6  (10.00) |  | 39.49 (10.06) | 11.05 (10.87) | 14.24 (9.70) | 14.20  (9.85) |  |
| LDH (U/L) |  |  |  |  | 0.245 |  |  |  |  | 0.866 |
| < 250 | 116 (75.82) | 25 (65.79) | 44 (80.00) | 47  (78.33) |  | 309.44 (78.81) | 81.64 (80.31) | 112.17 (76.40) | 115.63 (80.21) |  |
| ≥ 250 | 37 (24.18) | 13 (34.21) | 11 (20.00) | 13  (21.67) |  | 83.20 (21.19) | 20.01 (19.69) | 34.65 (23.60) | 28.54 (19.79) |  |
| No. of Metastatic lesions |  |  |  |  | 0.010 |  |  |  |  | 0.337 |
| 1-3 | 132 (86.27) | 31 (81.58) | 43 (78.18) | 58  (96.67) |  | 335.46 (85.44) | 82.19 (80.86) | 119.76 (81.57) | 133.51 (92.61) |  |
| 4-5 | 21 (13.73) | 7  (18.42) | 12 (21.82) | 2  (3.33) |  | 57.18 (14.56) | 19.46 (19.14) | 27.06 (18.43) | 10.66  (7.39) |  |
| Property |  |  |  |  | 0.604 |  |  |  |  | 0.929 |
| Osteogenesis | 87 (56.86) | 19 (50.00) | 32 (58.18) | 36  (60.00) |  | 232.74 (59.28) | 63.14 (62.11) | 86.24 (58.74) | 83.36 (57.83) |  |
| Osteolysis | 66 (43.14) | 19 (50.00) | 23 (41.82) | 24  (40.00) |  | 159.90 (40.72) | 38.51 (37.89) | 60.58 (41.26) | 60.80 (42.17) |  |
| PCT Regimen |  |  |  |  | 0.171 |  |  |  |  | 0.596 |
| TPF | 44 (28.76) | 5  (13.16) | 19 (34.55) | 20  (33.33) |  | 106.16 (27.04) | 19.09 (18.78) | 47.31 (32.22) | 39.77 (27.58) |  |
| TP | 39 (25.49) | 9  (23.68) | 11 (20.00) | 19  (31.67) |  | 107.35 (27.34) | 25.73 (25.32) | 28.65 (19.52) | 52.96 (36.74) |  |
| PF | 47 (30.72) | 14 (36.84) | 18 (32.73) | 15  (25.00) |  | 123.52 (31.46) | 39.41 (38.77) | 48.79 (33.23) | 35.32 (24.50) |  |
| GP | 9  (5.88) | 4  (10.53) | 3  (5.45) | 2  (3.33) |  | 19.17 (4.88) | 7.38 (7.26) | 9.02 (6.14) | 2.78  (1.93) |  |
| Multiple or Other Regimes | 14  (9.15) | 6  (15.79) | 4  (7.27) | 4  (6.67) |  | 36.43 (9.28) | 10.04 (9.88) | 13.05 (8.89) | 13.33  (9.25) |  |
| Response after PCT |  |  |  |  | 0.812 |  |  |  |  | 0.690 |
| PR | 109 (71.24) | 28 (73.68) | 40 (72.73) | 41  (68.33) |  | 278.67 (70.97) | 67.64 (66.54) | 102.19 (69.60) | 108.84 (75.50) |  |
| PD/SD | 44 (28.76) | 10 (26.32) | 15 (27.27) | 19  (31.67) |  | 113.96 (29.03) | 34.01 (33.46) | 44.63 (30.40) | 35.33 (24.50) |  |
| Administration of CCT |  |  |  |  | NA |  |  |  |  | NA |
| No | 99 (64.71) | 38 (100.00) | 32 (58.18) | 29  (48.33) |  | 288.74 (73.54) | 101.65(100.0) | 97.33 (66.29) | 89.76 (62.26) |  |
| Yes | 54 (35.29) | 0  (0.00) | 23 (41.82) | 31  (51.67) |  | 103.90 (26.46) | 0.00 (0.00) | 49.49 (33.71) | 54.41 (37.74) |  |

**Table S5** Detailed results among three treatment modalities in two risk group.

|  | Low-risk | | | | High-risk | | | |
| --- | --- | --- | --- | --- | --- | --- | --- | --- |
|  | 3-year OS | 5-year OS | HR (95%CI) | P value | 3-year OS | 5-year OS 89. | HR  (95%CI) | P value |
| PCT + LRRT vs. PCT | 86.2%  vs. 84.3% | 82.1%  vs. 66.4% | 0.71  (0.25-2.01) | 0.520 | 65.9%  vs. 51.3% | 43.6%  vs. 25.7% | 0.57  (0.30-1.07) | 0.076 |
| PCT + LRRT + bRT vs. PCT + LRRT | 94.3%  vs. 86.2% | 87.7%  vs. 82.1% | 0.62  (0.22-1.76) | 0.362 | 77.4%  vs. 65.9% | 53%  vs. 43.6% | 0.61  (0.33-1.15) | 0.124 |
| PCT + LRRT + bone RT vs. PCT | 94.3%  vs. 84.3% | 87.7%  vs. 66.4% | 0.63  (0.36-1.11) | 0.101 | 77.4%  vs. 51.3% | 53%  vs. 25.7% | 0.58  (0.41-0.82) | 0.001 |
| Overall comparison | -- | -- | 0.65  (0.37-1.14) | 0.309 | -- | -- | 0.59  (0.42-0.83) | 0.007 |

Note: All analyses were conducted using the Kaplan-Meier survival curves and log-rank test.
